# Difficulties in auditory organization as a cause of reading backwardness? An auditory neuroscience perspective

**DOI:** 10.1111/desc.12457

**Published:** 2016-09-22

**Authors:** Victoria Leong, Usha Goswami

**Affiliations:** ^1^ Centre for Neuroscience in Education Department of Psychology University of Cambridge UK; ^2^ Division of Psychology Nanyang Technological University Singapore

## Abstract

Over 30 years ago, it was suggested that difficulties in the ‘auditory organization’ of word forms in the mental lexicon might cause reading difficulties. It was proposed that children used parameters such as rhyme and alliteration to organize word forms in the mental lexicon by acoustic similarity, and that such organization was impaired in developmental dyslexia. This literature was based on an ‘oddity’ measure of children's sensitivity to rhyme (e.g. wood, *book*, good) and alliteration (e.g. sun, sock, *rag*). The ‘oddity’ task revealed that children with dyslexia were significantly poorer at identifying the ‘odd word out’ than younger children without reading difficulties. Here we apply a novel modelling approach drawn from auditory neuroscience to study the possible sensory basis of the auditory organization of rhyming and non‐rhyming words by children. We utilize a novel Spectral‐Amplitude Modulation Phase Hierarchy (S‐AMPH) approach to analysing the spectro‐temporal structure of rhyming and non‐rhyming words, aiming to illuminate the potential acoustic cues used by children as a basis for phonological organization. The S‐AMPH model assumes that speech encoding depends on neuronal oscillatory entrainment to the amplitude modulation (AM) hierarchy in speech. Our results suggest that phonological similarity between rhyming words in the oddity task depends crucially on slow (delta band) modulations in the speech envelope. Contrary to linguistic assumptions, therefore, auditory organization by children may not depend on phonemic information for this task. Linguistically, it is assumed that ‘book’ does not rhyme with ‘wood’ and ‘good’ because the final phoneme differs. However, our auditory analysis suggests that the acoustic cues to this phonological dissimilarity depend primarily on the slower amplitude modulations in the speech envelope, thought to carry prosodic information. Therefore, the oddity task may help in detecting reading difficulties because phonological similarity judgements about rhyme reflect sensitivity to slow amplitude modulation patterns. Slower amplitude modulations are known to be detected less efficiently by children with dyslexia.

## Research highlights

- We apply a novel model of speech encoding based on the neuronal oscillatory hierarchy to the rhyme oddity task.
- We show that children's rhyme judgements depend primarily on auditory sensitivity to relatively slow amplitude envelope information.

## Introduction

Nursery rhymes and rhyming games are an ubiquitous part of childhood (Opie & Opie, 1987). As well as being fun, these games and nursery routines play a key role in children's development of ‘phonological awareness’, the ability to identify and manipulate different phonological units within words such as syllables, rhymes and phonemes (Bryant, Bradley, Maclean & Crossland, 1989). Phonological awareness in turn plays a critical developmental role in the acquisition of reading and spelling, across languages (Ziegler & Goswami, 2005). The causal connection between phonology and reading was established in part by two important studies carried out by Bradley and Bryant (1978, 1983), using an ‘oddity’ task based on rhyme and alliteration. In one study, they showed that 10‐year‐old children with reading difficulties were significantly poorer at identifying the odd word out than 7‐year‐old typically developing children (Bradley & Bryant, 1978). In a second study, they gave 4‐ and 5‐year‐old pre‐reading children a three‐item form of the oddity task (‘cot, pot, *hat*’; ‘*hill*, pin, pig’), and showed that individual differences in performance were a unique predictor of reading and spelling skills measured 4 years later (Bradley & Bryant, 1983). Training the poorest‐performing children in sound categorization was also shown to improve reading and spelling achievement. Although phonological awareness is now studied using many different tasks, at many different linguistic levels, the oddity task has played an important role in developmental studies across languages, and in establishing the causal link between phonological awareness and reading (Goswami, 2012, for a summary).

At the same time, Bradley and Bryant's original claim about *auditory* organization has been neglected. Instead, the focus has been on linguistically defined units of phonology such as phonemes, which do not have a simple correspondence to acoustic cues in the speech signal. In linguistics, one research focus has been on phonological ‘neighbourhoods’, an organization of the mental lexicon based on phonological similarity. Adult researchers define phonological neighbours using a one‐phoneme‐different criterion (e.g. for the target *weed*: neighbours would include *weep*,* need*,* wood*; Luce & Pisoni, 1998). Child research suggests that earlier in development, phonological neighbourhoods may be organized in terms of onset‐rime similarity rather than phonemic similarity (e.g. De Cara & Goswami, 2003). Nevertheless, the rhyme versions of the oddity task used by Bradley and Bryant (1978, 1983) depended on phonemic changes, as the rime of the odd word out in each trial differed by only one phoneme (*peel*, weed, need, deed). Accordingly, there has been debate about exactly what the oddity task measures, rhyme awareness or phoneme awareness (Bowey, 1994). It has also been argued that children may solve the oddity task on the basis of ‘global phonological similarity’, utilizing holistic representations of words that have little internal phonological structure (e.g. Carroll & Snowling, 2001). Carroll and Snowling (2001) argued that words can sound globally similar without sharing any phonemes, such as the words ‘beach’ and ‘dish’, which share phonetic features such as close front vowels.

Recent advances in auditory neuroscience enable a novel acoustic perspective on the oddity task and what it might be measuring. Research on neural speech encoding has revealed a central role for oscillatory neural networks in auditory cortex (Giraud & Poeppel, 2012; Poeppel, 2014, for recent summaries). These networks encode temporal modulations in speech at the speech‐relevant rates of *delta* (~1–3 Hz), *theta* (~4–8 Hz), *beta* (~15–30 Hz), and *low gamma* (~30–50 Hz) (see Poeppel, 2014). These different modulation rates appear to provide a basis for parsing the continuous speech signal into linguistically relevant units (e.g. *delta* – syllable stress patterns, *theta* – syllables, *beta* – onset‐rime units, *low gamma* – phonetic information; see Ghitza & Greenberg, 2009; Giraud & Poeppel, 2012; Leong & Goswami, 2015). The networks align their activity to energy patterns in the speech signal by phase‐re‐setting their activity on the basis of amplitude ‘rise times’. Amplitude rise times specify the rates of change of energy in the speech signal, enabling the temporal alignment of different oscillatory rhythms with different speech rhythms (phase alignment or neuronal *entrainment*; Giraud & Poeppel, 2012). Neuronal oscillations are known to be temporally nested in phase and power over different timescales, which maximizes the information about the speech signal extracted by the brain (Lakatos, Shah, Knuth, Ulbert, Karmos *et al*., 2005). Slower oscillatory activity (delta band activity, ~ 2 Hz) governs theta band oscillatory activity (~ 5 Hz), which in turn governs the faster oscillatory activity thought to encode phonetic information in the beta and low gamma bands (~ 35 Hz; Gross, Hoogenboom, Thut, Schyns, Panzeri *et al*., 2013). This feedforward entrainment is driven by the physical properties of the speech envelope, and for adults is also modulated by faster top‐down activity, which appears to be driven by context and expectation (Park, Ince, Schyns, Thut & Gross, 2015). There are no comparable neural studies for children.

Regarding children, discovery of the oscillatory hierarchy makes it timely to consider modelling the child‐directed speech signal in terms of AM patterns in the speech envelope. Accordingly, we have recently modelled the spectro‐temporal modulation structure of children's nursery rhymes (Leong & Goswami, 2015). We found that the speech signal for nursery rhymes contains hierarchically nested amplitude modulations at similar temporal rates to the neuronal oscillatory bands (delta, theta, beta, low gamma; see Leong, 2012; Leong & Goswami, 2015). Further, we found that *phase alignment* between the slower AM rates (delta and theta) was crucial for adult perception of the prosodic patterns in children's nursery rhymes (Leong, Stone, Turner & Goswami, 2014). Meanwhile, with respect to dyslexia, we found that children with dyslexia showed atypical oscillatory encoding in the neural delta band when listening to rhythmic speech (Power, Mead, Barnes & Goswami, 2013). The children showed an earlier preferred phase in delta, implying that the dyslexic brain phase‐aligns to less informative portions of the slower amplitude modulations in the speech signal. Phonologically, these slower modulations are thought to relate to prosodic information and syllabic parsing (Ghitza & Greenberg, 2009). Our S‐AMPH approach also enables us to analyse the spectro‐temporal structure of the rhyming and non‐rhyming items in the oddity task.

To assess the acoustic spectro‐temporal similarity of monosyllabic rhyming words, the S‐AMPH model generates a hierarchical representation of the dominant spectral (acoustic *frequency*) and temporal (oscillatory *rate*) modulation patterns in the speech envelope of each word (Leong & Goswami, 2015). The AM patterns at three temporal rates (centred on ~2 Hz, ~5 Hz and ~20 Hz) form a three‐tier nested hierarchy, which mirrors the linguistic phonological hierarchy of stressed syllables, syllables, and onset‐rime units in the original nursery rhyme corpus (see Leong & Goswami, 2015). Oscillatory cycles at each AM rate thus correspond to phonological units of different sizes. The number of bands and their respective bandwidths were originally determined using PCA dimensionality reduction of original high‐dimensional spectral (29 ERB‐spaced frequency channels spanning 100–7250 Hz) and temporal (24 modulation channels spanning 0.9–40 Hz) envelope representations. The three AM rates derived from the PCA cover frequency ranges that linguistically correspond approximately to ‘Stress’, ‘Syllable’ and ‘Onset‐Rime’ patterning in speech respectively.

Here we applied the S‐AMPH to the spoken stimuli from two rhyme oddity tasks that we had administered to children taking part in an ongoing study of the possible auditory basis of developmental dyslexia (Goswami, Mead, Fosker, Huss, Barnes *et al*., 2013). This enabled us to compare the output of the model with the phonological judgements made by the children. We first estimated the acoustic similarity of the temporal structure of rhyming versus non‐rhyming items using two different similarity metrics, (1) mutual information (MI, a nonlinear information theoretic measure based on conditional probability) and (2) magnitude squared coherence (MSC, a linear measure based on cross‐covariance). This allowed us to explore whether the acoustic correlates of ‘rhyme’ reside primarily in the temporal patterning of slower or faster speech modulations or both. As current linguistic models assume that faster temporal modulations encode the phonemic differences utilized in the oddity task, a priori it would be expected that the acoustic correlates of rhyme would occur largely at the fastest modulation rate. Note that the acoustic correlates of rhyme have important consequences for the *neural encoding* of rhyme by oscillatory entrainment as well as implications for the auditory organization of the child's mental lexicon by onset and rime.

**Figure 1:**
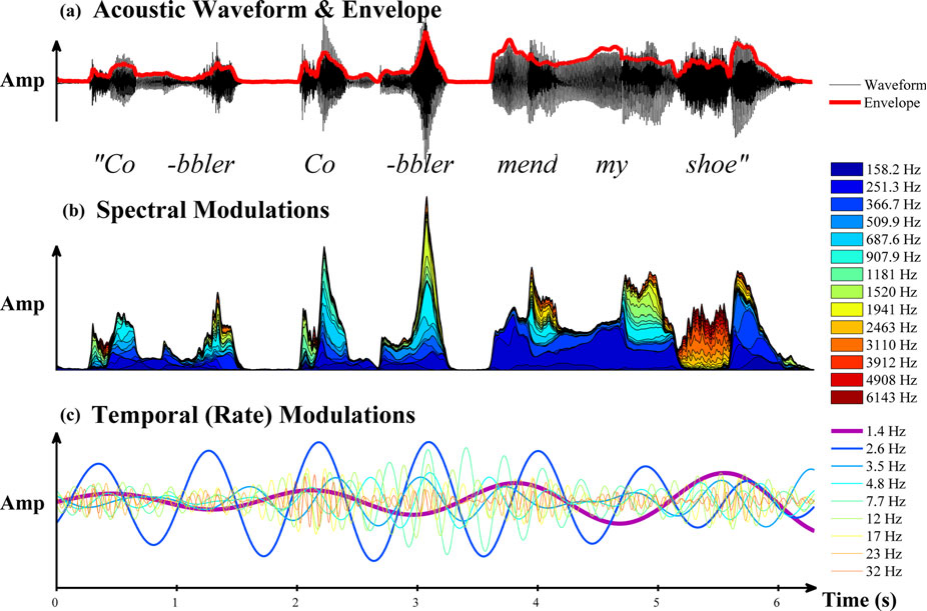
Spectro‐temporal modulation structure of a nursery rhyme sentence. Panel (a) shows the original acoustic waveform of the speech signal with the whole‐band amplitude envelope overlaid in bold. Panel (b) shows the spectral envelopes for the range of frequencies represented by the human cochlea (100–7250 Hz, ERB_n_‐spaced over 29 channels), with lower frequencies in blue and higher in red, plotted cumulatively (stacked, colour legend shows centre frequencies for alternate channels). Panel (c) shows a representative range of the modulation rates present in the wholeband envelope, log‐spaced between 0.9–40 Hz.

To illustrate how this modelling would represent the temporal information in children's nursery rhymes, the rich spectro‐temporal modulation structure that is present in the nursery rhyme sentence ‘Cobbler, cobbler, mend my shoe’ is shown in Figure 1. It is clear that most of the speech energy (the largest amplitude modulations) resides in the ‘Stress’ and ‘Syllable’ rates of the utterance (in this speech corpus, ~1.4 Hz [RMS = 2.7 × 10^−3^ units] and ~2.6 Hz [RMS = 6.1 × 10^−3^ units], respectively; see Leong, 2012). Notice also that each of the seven peaks in the ‘Syllable‐rate’ AM correspond to the occurrence of a single uttered syllable in the nursery rhyme.

## Methods

### Participants

A total of 101 children participated in the study: 40 children with dyslexia ([DY], 22 male, 18 female); 36 chronological age‐matched controls ([CA], 12 male, 24 female); and 25 reading‐level matched controls ([RL], 11 male, 14 female). The children were all taking part in a longitudinal study of developmental dyslexia (Goswami *et al*., 2013), and the data reported here were collected in Years 2–4 of the study, when the children with dyslexia were aged on average 9, 10 and 11 years, respectively. Children were recruited via learning support teachers, and only children who had no additional learning difficulties (e.g. dyspraxia, ADHD, autistic spectrum disorder, specific language impairment), a nonverbal IQ above 85, and English as the first language spoken at home were included. All children received a short hearing screen using an audiometer. Sounds were presented in both the left and right ear at a range of frequencies (250, 500, 1000, 2000, 4000, 8000 Hz), and all children were sensitive to sounds at 20 dB HL or less for both ears across all frequencies. Standardized measures of reading (British Ability Scales; Elliott, Smith & McCulloch, 1996) and IQ were administered (Wechsler Intelligence Scale for Children [WISC‐III]; Wechsler, 1992) and are shown in Table 1. For more detail on the sample, please see Goswami *et al*. (2013).

**Table 1:** Participant characteristics by group

	Dyslexic *N *=* *40	CA *N *=* *36	RL *N *=* *25	*F* (2, 98)
Age in years when study began^A^	8.6 (0.9)	8.5 (0.9)	6.8 (0.5)	41.9***
Reading age in years^B^	7.0 (1.2)	9.4 (1.8)	7.1 (0.8)	33.5***
*WISC FSIQ*	103.7 (13.1)	109.7 (11.3)	105.0 (10.8)	2.8
*Oddity Task Year 1, % correct* B	61.3 (17.7)	76.7 (16.9)	61.2 (17.0)	9.4***
*Oddity Task Year 2, % correct* B	67.6 (16.7)	85.6 (11.8)	70.8 (14.5)	15.4***
*Oddity Task Year 3, % correct* B	73.9 (14.7)	88.6 (8.9)	79.0 (15.7)	12.0***

*Note*: Standard deviations in parentheses. ***p <* *.0001

^A^CA = DYS > RL, ^B^RL = DYS < CA

### Rhyme oddity task

A rhyme oddity task was administered in years 2, 3 and 4 of the study, beginning at age 9 years (DY) or 7 years (RL). In Years 2 and 3, children listened to 20 trials of sets of three words comprising two rhyming words and one non‐rhyming word (e.g. ‘rod’, ‘nod’, ‘shop’). In Year 4, 10 triples were real words, while the other 10 triples were non‐words (e.g. ‘foss’, ‘noss’, ‘loff’). All the words were monosyllabic and had a simple CVC or CVCC structure. Within a word triple, the three words each began with a different onset but contained the same vowel nucleus. The two rhyming words shared the same coda, but the non‐rhyming word ended with a different coda. The stimuli were digitized speech created from a native female speaker of standard Southern British English, and presented by computer via headphones. The word triples were presented in one of three fixed randomized orders. Errors by word triple are shown in Appendix 1. Across groups, the lowest error proportion for any single word triple was 0 (‘wool’, ‘full’, ‘push’) and the highest error proportion was 0.55 (‘pike’, ‘like’, ‘ripe’).

### Phonological similarity modelling with the S‐AMPH

The S‐AMPH representation is obtained by a two‐stage filtering process. First, the raw acoustic signal is band‐pass filtered into five spectral bands using a series of adjacent finite impulse response (FIR) filters. These five bands are: (1) 100–300 Hz; (2) 300–700 Hz; (3) 700–1750 Hz; (4) 1750–3900 Hz; and (5) 3900–7250 Hz. Next, the Hilbert envelope is extracted from each of the five sub‐band filtered signals. These Hilbert envelopes are then passed through a second series of band‐pass filters in order to isolate the three different AM rate bands. These three AM rates are designated here the ‘Stress’ rate (0.9–2.5 Hz), ‘Syllable’ rate (2.5–12 Hz) and ‘Onset‐Rime’ rate (12–40 Hz). For a discussion of the derivation of the number of bands and bandwidths, please see Leong (2012). The result of this two‐step filtering process is a 5 × 3 spectro‐temporal representation of the speech envelope, made up of 15 AMs in total.

For each word triple in the total 40 triples, we then computed acoustic spectro‐temporal similarity metrics for the rhyming word pair (e.g. ‘nurse’–’verse’), and also the two non‐rhyming word pairs (e.g. ‘nurse’–’worth’ and ‘verse’–’worth’). It was expected that children would make fewer errors in rhyme judgement when the rhyming words within a word set showed high spectro‐temporal modulation similarity to each other, and low similarity to the non‐rhyming word. Accordingly, for each word set, we computed spectro‐temporal similarity metrics for the rhyming word pair as well as for the two non‐rhyming word pairs. Two different similarity metrics were used: (1) *mutual information* (MI) and (2) magnitude squared *coherence* (MSC). These two metrics operate on different assumptions and computational principles, as coherence is based on cross‐covariance and assumes a linear relationship whereas MI is a probabilistic measure that does not assume linearity. Therefore, if both metrics were to yield similar results, the results are likely to be an accurate reflection of the stimuli, rather than artefacts of the computational process. Note that such data would indicate the primary spectro‐temporal acoustic bases contributing to the phonological similarity of rhyming words, not the *only* spectro‐temporal acoustic bases. For example, if the modelling showed that slower AM information was of primary importance, this would not mean that children may not also use faster AM information in making particular judgements.

Mutual information indices quantify the degree of predictability between two signals (i.e. conditional probability). In the current context, mutual information can be thought of as the reduction in uncertainty about the spectro‐temporal pattern of the word ‘nurse’ that can be gained from observing the spectro‐temporal pattern of the word ‘verse’. Thus, rhyming words with more similar spectro‐temporal patterning should be associated with a greater reduction in uncertainty (higher mutual information) than non‐rhyming words with less similar spectro‐temporal patterning. The magnitude squared coherence metric measures the degree of coupling or temporal alignment between two spectro‐temporal envelopes at specific modulation rates. To maximize the temporal alignment between the envelopes of the words being compared, their envelopes were re‐sampled to the same length and then *z*‐scored. Resampling to the same length was used to ensure that differences in sample duration would not artificially reduce the computed index of phonological similarity. For example, two tokens of the identical word spoken rapidly versus slowly will have a lower acoustic similarity than expected, simply because their AMs are temporally misaligned, even though the tokens are phonologically identical. The purpose of *z*‐scoring was to standardize the mean and variance between different samples (e.g. accounting for differences in loudness). The coherence and MI metrics were then computed using these re‐sampled and *z*‐scored envelopes.

The *magnitude squared coherence* metric MSC(f) was the *cross‐spectrum* between the two envelopes, C_xy_(f); the Fourier transform of the cross‐covariance function, normalized by the product of the autospectra of each envelope, A_xx_(f) and A_yy_(f):

$$\text{MSC}\text{(f)}=|C_{\mathrm{xy}}{\left(f\right)|}^{2}/A_{\mathrm{xx}}\left(f\right)*A_{\mathrm{yy}}\left(f\right)$$

If envelopes *x* and *y* are identical, then their auto power spectra would be equal to each other, and also equal to their cross‐spectrum. In this case, their computed coherence would take the maximum value of 1. On the other hand, if envelopes *x* and *y* are completely unrelated, their cross‐spectrum would be zero for all frequencies, yielding the minimum coherence value of 0. Large values in the cross‐spectrum can result either from strong alignment between the two envelopes (i.e. they have the same ‘shape’) or from high power in one or both envelopes at particular frequencies. This is a potential confound when the cross‐correlation alone is used. However, as the coherence metric is normalized by the autospectra of the two envelopes, this effectively isolates the effect of coupling (temporal alignment), minimizing the confound of differences in modulation power at different frequencies. The power at each frequency was computed using Welch's averaged modified periodogram, and the maximum MSC value within each AM range was computed.

*Mutual information* was computed using the Matlab Information Breakdown Toolbox (Magri, Whittingstall, Singh, Logothetis & Panzeri, 2009). Raw MI values were computed for each pair of rhyming and non‐rhyming words between their 15 corresponding AMs by discretizing and binning the re‐sampled and *z*‐scored amplitude value at each timepoint into four bins. These four bins were symmetrically spaced about the mean (μ  =  0) to ensure that each bin was well populated with observations (bin edges = −2.5, −0.5, 0, 0.5, 2.5). For each pair of corresponding AMs (X and Y), the raw MI value was computed as:

$$I\left(\text{X; Y}\right)=\sum_{x=1}^{15}\sum_{y=1}^{15}p\left(\text{x,y}\right)\;\;\text{log}\left(p\left(x,y\right)/p\left(x\right)p\left(y\right)\right)$$

**Figure 2:**
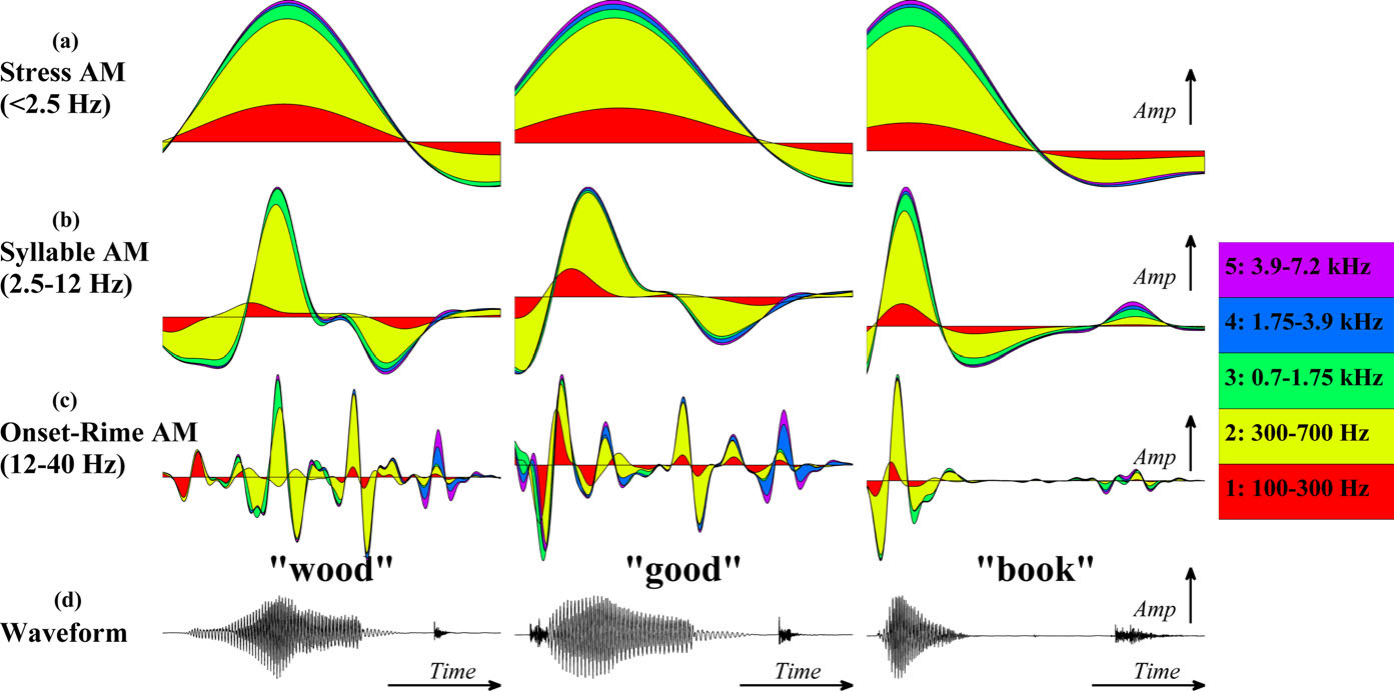
*S‐* AMPH spectro‐temporal modulation patterns for words in an oddity trial. The triple ‘wood, good, book’ is used to illustrate the amplitude modulation hierarchy comprising (a) ‘Stress’ (< 2.5 Hz), (b) ‘Syllable’ (2.5–12 Hz) and (c) ‘Onset‐Rime’ (12–40 Hz) tiers. AM hierarchies are derived for each of 5 acoustic frequency bands spanning 100 to 7250 Hz. Notice that the ‘Stress’ AM of rhyming and non‐rhyming words all share a similar overall shape and energy distribution across the five frequency bands, but they are distinguished by their *phase* pattern, where rhyming words ‘wood’ and ‘good’ show a later peak than non‐rhyming word ‘book’. Panel (d) shows the sound pressure waveform of the original speech signal.

Raw MI values were first corrected downward for bias using the quadratic extrapolation procedure (Strong, Koberle, de Ruyter van Steveninck & Bialek, 1998) and subsequently by shuffling, which destroyed the temporal correspondence between AM pairs (100 iterations per AM pair). The mean shuffled MI value was subtracted from the raw MI value giving the final corrected MI value. Bias correction is necessary because the MI estimation procedure is based on the assumption that signals are of an infinite length. If signals are not of an infinite length, the process will overestimate the true MI value. To illustrate how the S‐AMPH would represent the spectro‐temporal information in the oddity triple ‘good, wood, book’, a schematic depiction is presented in Figure 2. Figure 2 shows that rhyming words such as ‘good’ and ‘wood’ contain very similar spectro‐temporal patterning, particularly at the slower ‘Stress’ and ‘Syllable’ AM rates. In particular, while the ‘Stress’ AM of the rhyming and non‐rhyming words shows a similar overall shape and energy distribution across the five frequency bands, the items are distinguished by their *phase* pattern, with the rhyming words ‘wood’ and ‘good’ showing a later peak than the non‐rhyming word ‘book’. The non‐rhyme ‘book’ also shows an absence of energy in the faster ‘Onset‐Rime’ modulation band at the end of the word relating to the lack of voicing. The statistical analyses reported next aim to elucidate which of these potential auditory cues (and other potential cues) most consistently distinguish rhyming and non‐rhyming words across the entire corpus of word triples.

## Results

**Figure 3:**
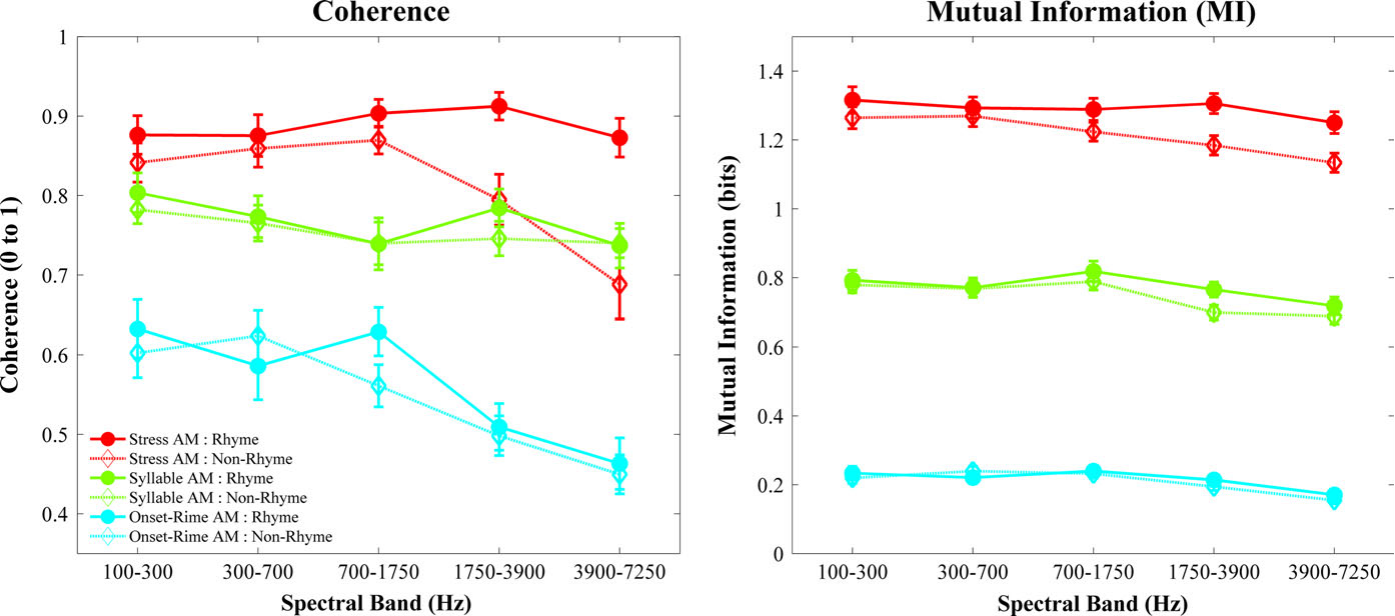
Mean acoustic spectro‐temporal modulation similarity between words as measured by the coherence metric and the mutual information metric (MI). In both plots, the x‐axis shows the five spectral bands (100–300 Hz, 300–700 Hz, 700–1750 Hz, 1750–3900 Hz, 3900–7250 Hz). The three AM rates are plotted in separate colours (‘Stress’ AM red, ‘Syllable’ AM green, ‘Onset‐Rime’ AM blue). Scores for rhyming word pairs are shown with bold lines and filled circles, scores for non‐rhyming word pairs (averaged for both pairs) are shown with dotted lines and hollow triangles. Error bars indicate the standard error.

In each of the three years of testing, the children with dyslexia showed a significant impairment in rhyme identification compared to their CA peers, shown in Table 1. However, they did not differ in rhyme sensitivity from the RL‐matched children in any year, despite having two more years of oral language experience. Remarkably, Coherence and MI analyses showed a highly similar pattern regarding the spectro‐temporal information in rhyming and non‐rhyming words, shown in Figure 3. To test whether there were statistical differences in the acoustic spectro‐temporal similarity of rhyming versus non‐rhyming word pairs, the Coherence and MI scores were used respectively as the dependent variable in two separate repeated measures ANOVAs with factors of Rhyme [2 levels: Rhyme vs Non‐Rhyme], AM Rate [3 levels: Stress, Syllable, Onset‐Rime] and Spectral Band [5 levels: Bands 1–5]. A significant interaction between rhyme status and AM rate would imply that different AM rates make differing acoustic contributions to perceived rhyme similarity. For both Coherence and MI metrics, the ANOVAs revealed a near significant main effect of Rhyme (Coh: *F*(1, 39) = 3.36, *p *=* *.074, *ηρ²* = 0.08; MI: *F*(1, 34) = 2.56, *p *=* *.12, *ηρ²* = 0.07), with rhyming word pairs showing higher scores than non‐rhyming word pairs. There were significant main effects of AM Rate (Coh: *F*(2, 78) = 193.2, *p *<* *.0001, *ηρ²* = 0.83; MI: *F*(2, 68) = 366.5, *p *<* *.0001, *ηρ²* = 0.92) with scores highest for the ‘Stress’ AM and lowest for the ‘Onset‐Rime’ AM, and of Spectral Band (Coh: *F*(4, 156) = 10.28, *p *<* *.0001, *ηρ²* = 0.21; MI: *F*(4, 136) = 6.10, *p *<* *.001, *ηρ²* = 0.15), with scores decreasing from the *lowest* frequency band (1) to the highest frequency band (5). The interaction between Rhyme and Spectral Band was non‐significant in the Coherence ANOVA, *F*(4, 156) = 1.90, *p *=* *.11, but significant in the MI ANOVA, *F*(4, 136) = 3.19, *p *=* *.015. Tukey post‐hoc analysis of this latter interaction showed that rhyming and non‐rhyming words differed only in Spectral Band 4 (*p *<* *.05). Importantly, *both* ANOVAs showed a significant interaction between Rhyme and AM Rate, MI (*F*(2, 68) = 3.31, *p *<* *.05, *ηρ²* = 0.09); Coherence (*F*(2, 78) = 4.90, *p *<* *.01, *ηρ²* = 0.11). In both cases, Tukey's post‐hoc tests revealed a significant difference between rhyming and non‐rhyming words at the slowest ‘Stress’ AM rate only (*p *<* *.01 for both metrics). This captures the different phase patterns visible in Figure 2. This acoustic analysis of phonological similarity suggests that rhyming and non‐rhyming word pairs differed primarily in their perceived similarity because of acoustic information at the *slowest* modulation timescale (the ‘Stress’ AM rate, 0.9–2.5 Hz). Meanwhile, the main effect of spectral band showed that the lowest frequency bands contributed most to phonological similarity (these spectral bands contribute largely to F0 and vowel perception).

Also of interest in this study was whether the acoustic similarity parameters identified by the modelling would be related to children's phonological performance. To assess whether the spectro‐temporal similarity of each word triple as measured by our AM approach would predict significant variance in the pattern of children's errors, we performed a single‐trial analysis. The aim was to see which modulation rates and spectral bands were the best predictors of individual differences in children's performance in the oddity task. For this analysis, we scored the proportion of errors observed for each of the 20 word triples that were presented in each year (shown as Appendix 1). For each word triple, the proportion of errors was computed as the total number of errors for each group divided by the total number of children in that group. This error scoring yielded values from 0 (no children made errors on this word triple) to 1 (all children made errors on this word triple). It was expected that children would make fewer errors in rhyme judgement when the rhyming words within a word set showed high spectro‐temporal modulation similarity to each other, and low similarity to the non‐rhyming word (i.e. a large rhyme vs non‐rhyme differential). Accordingly, we took the Coherence and MI *difference scores* (rhyme score minus mean of two non‐rhyme scores) for each word triple and correlated these difference scores to the errors made by the 101 participating children. Using the difference scores also had the added benefit of correcting for variations in mean similarity across different word sets. The distributions of error proportions for all three groups of children were normal. As would be expected, both Coherence and MI difference scores produced negative correlations (i.e. a *greater* rhyme/non‐rhyme differential resulted in *lower* rates of error). The correlations across the three bands and groups are shown in Table 2 (Benjamini‐Hochberg [1995] FDR‐corrected for multiple comparisons). All children showed significant correlations for slow, low‐frequency spectro‐temporal patterns only (‘Stress’ AM patterns within Spectral Bands 1 and 2 [100–700 Hz]).

**Table 2:** Correlation coefficients between children's errors in performance, and differences in Coherence (dCoh) and mutual information (dMI) scores for rhyming vs. non‐rhyming words. FDR‐corrected significant correlations are highlighted. Frequency bands: (1) 100–300 Hz; (2) 300–700 Hz; (3) 700–1750 Hz; (4) 1750–3900 Hz; (5) 3900–7250 Hz

		DYs		CAs		RLs	
		dCoh	dMI	dCoh	dMI	dCoh	dMI
‘Stress’ AM	Band 1	−.44*	−.31	−.50*	−.40	−.47*	−.37
	Band 2	−.33	−.39	−.41*	−.43*	−.46*	−.45*
	Band 3	−.25	−.16	−.13	−.07	−.29	−.13
	Band 4	−.31	.13	−.15	.21	−.40*	−.04
	Band 5	−.31	−.14	−.16	.15	−.36	−.04
‘Syllable’ AM	Band 1	−.13	−.07	−.17	−.18	−.15	−.14
	Band 2	−.30	−.14	−.30	−.08	−.24	−.15
	Band 3	−.27	−.27	−.19	−.34	−.22	−.30
	Band 4	.10	.06	.12	.09	.06	.09
	Band 5	−.01	−.13	.01	.05	−.12	.09
‘Onset‐Rime’ AM	Band 1	.03	.18	.05	.07	−.08	.10
	Band 2	−.22	−.19	−.20	−.04	−.16	−.11
	Band 3	−.10	−.21	.14	−.09	−.14	−.14
	Band 4	−.05	−.08	.15	.08	−.02	−.24
	Band 5	−.21	−.04	−.12	−.01	−.33	−.06

* FDR‐corrected *p *<* *.05.

Finally, we assessed the proportion of variance in children's performance that was predicted by variations in spectro‐temporal similarity across word triples. Again, we used difference scores as the DV in these analyses, rather than raw values. We used either Coherence or MI difference scores to compute three multiple regression equations, respectively, taking CA errors, DY errors and RL errors as the DV in each case. As we expected the AM patterns to be highly inter‐correlated for the same word (especially between adjacent spectral bands), this could result in multicollinearity in our regression analyses. Therefore, prior to performing the regression, we first performed a factor analysis using the principal components extraction method on the 15 AM variables to remove intercorrelations.

### Coherence

The factor analysis extracted four factors which collectively accounted for 70.5% of variance across the 15 spectro‐temporal AM variables. The factor loadings are shown in Table 3, and correspond well to the spectral banding structure used in the S‐AMPH model. For example, Factor 1 (explaining 37.7% of variance) loaded most strongly onto the lowest spectral bands 1 and 2, across ‘Stress’, ‘Syllable’ and ‘Onset‐Rime’ AMs. Factor 2 (12.8% variance) loaded most strongly onto spectral bands 3 and 4 for the ‘Syllable’‐ and ‘Onset‐Rime’‐rate AMs. Factor 3 (12.3% variance) loaded most strongly onto spectral bands 4 and 5 for the ‘Stress’ AM only, and Factor 4 (7.7% variance) loaded most strongly onto spectral band 5 for the ‘Syllable’ AM only. These four de‐correlated factors were then entered into each regression equation (one equation for CA errors, one for RL errors, one for DY errors).

**Table 3:** Varimax‐normalized factor loadings for the first four factors, collectively accounting for 70.5% of spectro‐temporal AM variance (Coherence difference metric). Loadings ≥ 0.7 are in bold. Frequency bands: (1) 100–300 Hz; (2) 300–700 Hz; (3) 700–1750 Hz; (4) 1750–3900 Hz; (5) 3900–7250 Hz

		Factor 1 (37.7%)	Factor 2 (12.8%)	Factor 3* (12.3%)	Factor 4 (7.7%)
‘Stress’ AM	Band 1	**0.71**	−0.17	0.49	−0.02
	Band 2	0.64	−0.06	0.60	−0.00
	Band 3	0.58	0.37	0.44	−0.30
	Band 4	0.18	0.23	**0.85**	−0.09
	Band 5	0.03	−0.16	**0.82**	0.28
‘Syllable’ AM	Band 1	**0.75**	−0.08	−0.06	0.41
	Band 2	0.69	0.03	0.32	0.38
	Band 3	**0.70**	0.38	0.25	0.12
	Band 4	0.21	**0.74**	0.04	0.35
	Band 5	0.25	0.25	0.24	**0.78**
‘Onset‐Rime’ AM	Band 1	**0.77**	0.17	−0.14	0.05
	Band 2	**0.78**	0.21	0.04	0.01
	Band 3	−0.05	**0.73**	0.11	0.15
	Band 4	0.20	**0.76**	0.01	−0.21
	Band 5	−0.04	0.35	0.58	0.21

* Significant contributor to children's errors.

### MI

Based on a scree plot, the first four factors extracted for the factor analysis collectively accounted for 63.1% of variance across the 15 spectro‐temporal AM variables. The factor loadings are shown in Table 4. Factor 1 (explaining 31.5% of variance) loaded most strongly onto spectral band 3 of the ‘Syllable’ AM and spectral band 1 of the ‘Onset‐Rime’ AM. Factor 2 (12.7% variance) loaded most strongly onto the lowest two spectral bands (1 and 2) of the ‘Stress’ AM. Factor 3 (9.9% variance) loaded most strongly onto the highest two spectral bands (4 and 5) of the ‘Stress’ AM only, and Factor 4 (9.0% variance) loaded most strongly onto spectral band 1 for the ‘Syllable’ AM only. These four de‐correlated factors were then entered into each regression equation (one equation for CA errors, one for RL errors, one for DY errors).

**Table 4:** Varimax‐normalized factor loadings for the first six factors, collectively accounting for 77.2% of spectro‐temporal AM variance (MI difference metric). Loadings ≥ 0.7 are in bold. Frequency bands: (1) 100–300 Hz; (2) 300–700 Hz; (3) 700–1750 Hz; (4) 1750–3900 Hz; (5) 3900–7250 Hz

		Factor 1 (31.5%)	Factor 2* (12.7%)	Factor 3 (9.9%)	Factor 4 (9.0%)
‘Stress’ AM	Band 1	0.13	**0.91**	0.23	0.04
	Band 2	0.11	**0.90**	0.10	0.11
	Band 3	0.66	0.30	0.16	−0.21
	Band 4	0.07	0.09	**0.73**	−0.08
	Band 5	−0.03	0.25	**0.80**	0.18
‘Syllable’ AM	Band 1	0.29	0.47	−0.15	**0.71**
	Band 2	0.48	0.24	−0.18	0.38
	Band 3	**0.85**	0.21	−0.02	0.03
	Band 4	0.04	0.20	−0.15	0.12
	Band 5	0.28	−0.07	0.32	0.37
‘Onset‐Rime’ AM	Band 1	**0.81**	−0.06	0.01	0.23
	Band 2	0.16	0.23	0.11	0.03
	Band 3	0.52	0.07	0.08	−0.27
	Band 4	0.10	−0.10	0.01	0.13
	Band 5	−0.04	−0.04	0.27	0.68

* Significant contributor to children's errors.

A forward stepwise selection format allowed automatic selection of the strongest predictors for each equation. The results are shown in Table 5.

**Table 5:** Forward stepwise regression models for Coherence and MI metrics for each group

Group	ANOVA	Adjusted *R* ^2^	Predictors in model	Β	*p*‐value
Coh					
CA	*F*(3, 36) = 3.41, *p *<* *.05	.156	Factor 3	−.03	.06
			Factor 1	−.03	.08
			Factor 2	.02	.08
DY	*F*(2, 37) = 4.64, *p *<* *.05	.157	Factor 3	−.05	<.05
			Factor 1	−.02	.15
RL	*F*(2, 37) = 7.17, *p *<* *.01	.240	Factor 3	−.05	<.01
			Factor 1	−.02	.16
MI					
CA	*F*(3, 32) = 5.29, *p *<* *.05	.202	Factor 2	−.04	<.01
			Factor 3	.02	.15
DY	*F*(1, 33) = 4.71, *p *<* *.05	0.098	Factor 2	−.04	<.05
RL	*F*(1, 33) = 6.01, *p *<* *.05	0.128	Factor 2	−.04	<.05

### Coherence

Across all three equations for all three groups of children, factor 3 (high‐frequency slow AMs) and factor 1 (low‐frequency AMs across all timescales) emerged as the strongest predictors of children's errors across the three participant groups. For the CA group, the final regression model (*F*(3, 36) = 3.41, *p *<* *.05) accounted for 15.6% of variability in performance (adjusted *R* ^2^), and included three factors: factor 3 (β  =  −.03, *p *=* *.06), factor 1 (β  =  −.03, *p *=* *.08), and factor 2 (β  =  .02, *p *=* *.08). For the DY group, the final regression model (*F*(2, 37) = 4.64, *p *<* *.05) accounted for 15.7% of variance, and included only factor 3 (β  =  −.05, *p *<* *.05) and factor 1 (β  =  −.02, *p *=* *.15). For the RL group, the final regression model (*F*(2, 37) = 7.17, *p *<* *.01) accounted for a robust 24.0% of variance and, similar to dyslexic children, only comprised factor 3 (β  =  −.05, *p *<* *.01) and factor 1 (β  =  −.02, *p *=* *.16). Therefore, while none of the spectro‐temporal factors for Coherence individually accounted for a significant proportion of the errors made by the CA children (factor 3 was closest, at *p *=* *.06), significant variance in the phonological errors made by children with dyslexia and their younger RL controls was consistently accounted for by factor 3 (slow AM information in spectral bands 4 and 5: slowly‐changing high‐frequency information).

### MI

Across all three equations for all three groups of children, factor 2 (corresponding to the ‘Stress’ AM in spectral bands 1 and 2) emerged as the *sole significant predictor* of children's errors. For the CA group, the final regression model (*F*(3, 32) = 5.29, *p *<* *.05) accounted for 20.2% of variability in performance (adjusted *R* ^2^), and included two factors: factor 2 (β  =  −.04, *p *<* *.01), and factor 3 (β  =  .02, *p *=* *.15). For the DY group, the final regression model (*F*(1, 33) = 4.71, *p *<* *.05) accounted for 9.8% of variance, and included just factor 2 (β  =  −.04, *p *<* *.05). For the RL group, the final regression model (*F*(1, 33) = 6.01, *p *<* *.05) accounted for 12.8% of variance and also comprised only factor 2 (β  =  −.04, *p *<* *.05). Therefore, Factor 2 (slow AM information in spectral bands 1 and 2; slowly changing low‐frequency information) was the strongest predictor of performance for all participants. Accordingly, variations in spectro‐temporal similarity across word triples as computed by the S‐AMPH model are consistent in identifying *slower modulations* (delta band, ‘Stress’ AM) in both low‐frequency (spectral bands 1 and 2; MI analyses) and high‐frequency (spectral bands 4 and 5; Coherence analyses) regions of the speech signal as playing a significant role in the errors made in the rhyme oddity task by children.

## Discussion

Individual differences in awareness of rhyme as measured by the oddity task predict the acquisition of reading across languages (e.g. Bradley & Bryant, 1983; Wimmer, Landerl & Schneider, 1994), and rhyme awareness is impaired in children with developmental dyslexia (Bradley & Bryant, 1978; Ziegler & Goswami, 2005). In their pioneering work, Bradley and Bryant suggested that rhyme and alliteration could be important ways of categorizing words by acoustic similarity for children. They proposed that the oddity task could measure the ‘auditory organization’ of spoken words in children's mental lexicons, assuming that such organization underpinned the development of phonological awareness. Here we investigated the possible nature of the acoustic cues that English‐speaking children use to organize their mental lexicons with respect to rhyme similarity, using a model derived from auditory neuroscience, the S‐AMPH (Leong, 2012; Leong & Goswami, 2015). Of interest were which acoustic parameters the model would identify as contributing most to auditory organization by rhyme, and whether these acoustic parameters would play a role in children's phonological judgements.

Accordingly, we first applied the model to each item in the oddity task, and then estimated the acoustic similarity between items using two different similarity metrics, MI and Coherence. Our analyses showed that the spectro‐temporal characteristics that made rhyming words acoustically similar and non‐rhyming words acoustically different in the oddity task were carried by slow amplitude modulations (delta band, ‘Stress’ AM) in the speech envelope. Further, when children's errors were examined, both the Coherence and MI metrics identified acoustic information at the *slowest* modulation timescale (the ‘Stress’ AM rate, 0.9–2.5 Hz) as consistently important. Rapid (here, beta and low gamma rate) modulations did not play a significant role in children's errors according to either metric. These findings are theoretically important. They suggest that the primary acoustic cues used by children for organizing the mental lexicon in terms of rhyme are the slower amplitude modulations that signify syllable structure, prosodic stress, and vowel identity. The more rapid modulations traditionally assumed to carry phonetic information in the speech signal did not contribute consistently to phonological similarity, at least for the oddity triplets analysed here.

In the larger neural literature, AM patterns in the delta band (‘Stress’ AM band, centred at 2 Hz) are primarily related to prosodic structure (Ghitza & Greenberg, 2009). Speech prosody carries speech rhythm, and children with dyslexia show reduced prosodic and rhythmic awareness (Goswami, Gerson & Astruc, 2010; Goswami *et al*., 2013). Further, a study of the role of acoustic sensitivity to 2 Hz *FM* in reading development by typically developing children also found this slower rate to be important for the development of phonological skills and reading (Talcott, Witton, Maclean, Hansen, Rees *et al*., 1999). Auditory neuroscience has shown that very slow rates of FM are encoded by the same neural populations that encode slow AM (Obleser, Hermann & Henry, 2012). Accordingly, new experimental assessments of the roles of ~2 Hz AM and FM in phonological development across languages may be valuable in terms of understanding the underlying neural mechanisms contributing to phonological development and auditory organization. Such investigations may also shed light on the acoustic difficulties contributing to developmental dyslexia.

The demonstration here that *monosyllabic* non‐rhyming words are primarily distinguished by spectro‐temporal differences in slow envelope information also provides converging evidence for the importance of sensitivity to amplitude rise time during children's phonological development. As noted earlier, neural networks in auditory cortex encode speech by re‐setting their endogenous oscillatory activity to be aligned temporally with amplitude modulations in the speech signal (Giraud & Poeppel, 2012). Amplitude rise times are particularly important for driving this neuronal phase‐resetting, acting as auditory ‘edges’ or cues to modulation rate that re‐set the oscillatory networks fluctuating at these rates (Gross *et al*., 2013; Doelling, Arnal, Ghitza & Poeppel, 2014). Sensitivity to amplitude envelope ‘rise time’ is impaired in children with dyslexia in many languages (English, French, Spanish, Chinese, Finnish, Dutch and Hungarian; see Goswami, 2011), and neuronal phase alignment to speech is atypical in English‐speaking children with dyslexia in the delta band (~2 Hz rate; Power *et al*., 2013). These developmental data suggest that amplitude rise times play an important role in neural speech encoding and phonological development. The current analyses suggest that slow AM information makes an important contribution to the perception of phonological similarities and differences between words at the rhyme level for all children.

Indeed, if we had used oddity stimuli with differing vowels as well as codas, the proportion of variance explained by slow AMs could have been much higher. Even so, our modelling data question the widespread convention in linguistics that phoneme‐based feature systems underlie phonological similarity, at least regarding children. In linguistic terms, the rimes of the words in the oddity tasks used here differed by a single *phoneme*. Indeed, the one‐phoneme‐different criterion is a widely accepted metric for quantifying phonological similarity relations (Luce & Pisoni, 1998). This metric has already been questioned in the field of child language acquisition (Dollaghan, 1994). For example, nursery rhymes like *Hickory, Dickory, Dock* rhyme ‘clock’ with ‘dock’ – yet these words are not similar according to phoneme‐based metrics, as they differ by two phonemes. Further, rhyme‐based rather than phoneme‐based organization of the mental lexicon by the pre‐reading child has been supported experimentally, at least for English (De Cara & Goswami, 2002, 2003). The acoustic spectro‐temporal similarity metrics introduced here thus offer a novel approach to analysing phonological development in terms of auditory organization, which would be applicable across languages.

In conclusion, our data suggest that sensitivity to slow (delta band or ‘Stress’) AMs in speech plays an important role in the auditory organization of the mental lexicon by rhyme by English‐speaking children. The two similarity metrics used here both revealed a significant interaction between rhyme status and AM rate, with significant acoustic differences between rhyming and non‐rhyming words carried by the slowest AM rate only (‘Stress’ AM, delta band). Regarding phonological rhyme judgements, the data revealed that children's errors were consistently related to slow delta band AM information, primarily in the lower frequency regions of the signal (100–700 Hz): Slow, low‐frequency acoustic information. Finally, the multiple regression analyses showed that the only significant variance in children's rhyming errors was accounted for by ‘Stress’ AM band information, slow energy variations in the delta band across almost all spectral regions (Bands 1, 2, 4 and 5; 100–700 Hz, and 1750–7250 Hz). Accordingly, sensitivity to slow amplitude modulation patterns in the speech envelope may play an important role in the development of phonological awareness. Further, the oddity task may be a useful cross‐language measure of phonological development precisely because oddity judgements rely primarily on accurate perception of slow envelope information.

## Appendix 1

Tables A1 and A2 show the word sets used for the Rime Oddity task in each year of testing (2, 3, 4), with their respective error proportions, listed by group. For ease of presentation, the two rhyming words in a given trial are listed first, followed by the non‐rhyming word (the correct answer). In the Rime Oddity test as presented to the children, the order of the rhyming and non‐rhyming words in each triple was randomized. Error proportions were computed by summing the total number of errors observed across the group, and dividing this by the number of children in that group. Possible values range from 0 (no errors from any children) to 1 (all children made an error).

**Table A1:** List of word triples used in test years 2 and 3 and their respective error proportions

Word sets	Average errors for Year 2 & Year 3		
	DY	CA	RA
dutch, hutch, budge	0.40	0.12	0.32
wag, nag, that	0.18	0.09	0.20
biz, fizz, give	0.26	0.13	0.26
pike, like, ripe	0.55	0.36	0.52
rib, fib, wig	0.34	0.30	0.36
knock, shock, jot	0.44	0.31	0.40
bird, gird, shirt	0.29	0.19	0.34
thick, nick, chit	0.47	0.25	0.46
rod, nod, shop	0.22	0.09	0.20
bud, mud, pup	0.23	0.10	0.28
cheek, meek, deed	0.32	0.12	0.26
lid, bid, rib	0.48	0.25	0.44
wake, shake, date	0.48	0.17	0.34
daze, gaze, case	0.25	0.07	0.24
gap, nap, jack	0.29	0.23	0.40
wish, fish, pith	0.38	0.25	0.34
wheat, cheat, meek	0.51	0.33	0.42
loss, moss, toff	0.37	0.19	0.38
dove, love, buzz	0.36	0.19	0.26
zip, nip, chick	0.52	0.07	0.38

**Table A2:** List of word triples used in test year 4 and their respective error proportions

Word sets	Errors for Year 4		
	DY	CA	RA
curl, hurl, fern	0.28	0.06	0.20
foss, noss, loff	0.33	0.17	0.28
wool, full, push	0.00	0.03	0.12
bish, mish, gith	0.28	0.14	0.16
mile, file, sign	0.18	0.11	0.24
chib, sib, nid	0.48	0.39	0.40
bird, third, merge	0.33	0.11	0.36
jud, fud, rup	0.33	0.17	0.32
lert, mert, sern	0.13	0.06	0.12
rizz, nizz, kiv	0.28	0.19	0.16
wood, good, book	0.28	0.06	0.20
like, bike, rice	0.15	0.08	0.12
hold, fold, post	0.20	0.06	0.08
nurse, verse, worth	0.23	0.14	0.24
fatch, satch, radge	0.35	0.11	0.20
touch, such, fudge	0.33	0.06	0.28
life, wife, hive	0.33	0.11	0.24
seff, heff, lep	0.30	0.14	0.12
farn, jarn, sarp	0.25	0.08	0.24
pung, vung, nuss	0.25	0.03	0.12
